# Co-existing Hypertension and Hyperhomocysteinemia Increases the Risk of Carotid Vulnerable Plaque and Subsequent Vascular Event: An MR Vessel Wall Imaging Study

**DOI:** 10.3389/fcvm.2022.858066

**Published:** 2022-03-30

**Authors:** Dongye Li, Huiyu Qiao, Xieqing Yang, Jin Li, Wei Dai, Xiaoyi Chen, Jun Shen, Xihai Zhao

**Affiliations:** ^1^Department of Radiology, Sun Yat-Sen Memorial Hospital, Sun Yat-Sen University, Guangzhou, China; ^2^Department of Biomedical Engineering, Center for Biomedical Imaging Research, Tsinghua University School of Medicine, Beijing, China; ^3^Department of Radiology, Beijing Chao-Yang Hospital, Capital Medical University, Beijing, China; ^4^Department of Neurology, Chinese People's Liberation Army (PLA) General Hospital, Beijing, China; ^5^Department of Radiology, Beijing Geriatric Hospital, Beijing, China

**Keywords:** homocysteine, hypertension, atherosclerosis, vulnerable plaque, stroke

## Abstract

**Purpose:**

This study sought to determine the associations of co-existing hypertension and hyperhomocysteinemia (H-Hcy) with carotid vulnerable plaque features and subsequent vascular events.

**Methods:**

Symptomatic patients with carotid atherosclerosis were enrolled and underwent carotid magnetic resonance (MR) vessel wall imaging. The patients were divided into the following groups: co-existing hypertension and H-Hcy group; isolated hypertension group; isolated H-Hcy group; and control group. The morphological and compositional characteristics of carotid plaques were assessed on MR images and compared among different groups. Univariate and multivariate cox regressions were used to calculate the hazard ratio (HR) and corresponding 95% confidence interval (CI) of co-existing hypertension and H-Hcy in predicting subsequent vascular events after at least 1-year followed-up.

**Results:**

In total, 217 patients (mean age, 59.4 ± 11.9 years; 154 males) were recruited. Patients in co-existing hypertension and H-Hcy group had a significantly higher prevalence of carotid lipid-rich necrotic core (LRNC) than isolated H-Hcy and control group (73.2 vs. 43.3 vs. 50%, *p* = 0.015). During the median follow-up time of 12.2 ± 4.3 months, 61 (39.8%) patients experienced vascular events. After adjusting for baseline confounding factors, co-existing hypertension and H-Hcy (HR, 1.82; 95% CI, 1.01–3.27; *p* = 0.044), presence of carotid LRNC (HR, 2.25; 95% CI, 1.09–4.65; *p* = 0.029), and combination of co-existing hypertension and H-Hcy and carotid LRNC (HR, 2.39; 95% CI, 1.26–4.43; *p* = 0.007) were significantly associated with subsequent vascular events.

**Conclusions:**

Co-existing hypertension and H-Hcy are associated with carotid vulnerable plaque features, such as LRNC. Combining co-existing hypertension and H-Hcy with carotid vulnerable plaque features has a stronger predictive value for subsequent vascular events than each measurement alone.

## Introduction

Ischemic stroke has become the leading cause of death in the Chinese population (1, 2). Carotid vulnerable plaques were demonstrated to be associated with cerebrovascular events (3). Many risk factors are associated with the initiation and progression of atherosclerotic disease (4, 5). Investigation of the critical factors for carotid vulnerable plaques is helpful in creating treatment strategies and preventing plaque disruption and subsequent cerebrovascular events.

Previous studies have shown that both hypertension (6) and elevated homocysteine (7, 8) in serum play a key role in the progression of atherosclerotic disease. It is reported that nearly 75% of Chinese patients with hypertension had hyperhomocysteinemia (H-Hcy), and such a condition has been defined as co-existing hypertension and H-Hcy (9). Previous studies demonstrated that co-existing hypertension and H-Hcy was associated with carotid plaque burden measured by computed tomographic angiography and ultrasound imaging (10). However, plaque burden is not an ideal indicator for vulnerable plaques and a substantial number of vulnerable features can be observed in patients with lower plaque burden (11). Multi-contrast vessel wall magnetic resonance (MR) imaging has been largely utilized to assess carotid vulnerable plaque compositional features validated by histology (12, 13), such as lipid-rich necrotic core (LRNC) and intraplaque hemorrhage (IPH). The relationship between co-existing hypertension and H-Hcy and carotid vulnerable compositional features determined by MR vessel wall imaging has not been studied. Increasing evidence has shown that both co-existing hypertension and H-Hcy and carotid vulnerable plaque compositional features could predict subsequent events (14, 15). We hypothesized that combined co-existing hypertension and H-Hcy with carotid vulnerable plaque may have a stronger predictive value for subsequent vascular events than each measurement alone. However, the incremental value of the combination of co-existing hypertension and H-Hcy with carotid vulnerable plaque features for future vascular events remains unknown.

This study sought to determine the associations of co-existing hypertension and H-Hcy with carotid vulnerable compositional features and subsequent vascular events utilizing the multi-contrast MR vessel wall imaging.

## Materials and Methods

### Data Sharing

The data that support the findings of this study are available from the corresponding author upon reasonable request.

### Study Population

In this study, patients who had recent cerebrovascular symptoms [ischemic stroke or transient ischemic attack (TIA) in the anterior circulation within 2 weeks] and carotid atherosclerosis detected using ultrasound were consecutively enrolled and underwent multi-contrast MR vessel wall imaging for extracranial carotid arteries. The exclusion criteria included (1) non cerebrovascular stroke (cardiogenic stroke, hemorrhagic stroke, etc.); (2) other vascular diseases (dissection and vasculitis); (3) history of radiation therapy in the neck; (4) pregnancy; (5) patients with severe disturbance of consciousness (coma, etc.); (6) patients with temporary H-Hcy due to a vitamin deficiency; and (7) contraindication to MR imaging examination. The MR imaging was performed within 2 weeks after the onset of symptoms. Clinical characteristics include age, sex, body mass index, smoking, diastolic and systolic blood pressure, serum homocysteine, hyperlipidemia, serum lipid levels of high-density lipoprotein (HDL), low-density lipoprotein (LDL), total cholesterol (TC), and triglyceride (TG), history of diabetes mellitus, and history of coronary heart disease were collected from the medical record. The information on the treatment of antihypertension, lipid-lowering, anticoagulation, and antiplatelet was also recorded. Hypertension was recorded as having the previous history of hypertension or systolic blood pressure equal to or higher than 140 mmHg and/or diastolic blood pressure equal to or higher than 90 mmHg for two times in the quiet condition. Hyperlipidemia was defined as fasting total cholesterol >2.3 mmol/L (220 mg/dL) or triglyceride >1.70 mmol/L (150 mg/dL). Diabetes mellitus was diagnosed based on fasting blood glucose >7.0 mmol/L (126 mg/dL) or 2-h post-prandial blood glucose >11.1 mmol/L (200 mg/dL). The H-Hcy was defined as serum homocysteine >15 μmol/L (16). The study protocol was approved by the institutional review board and a written consent form was obtained from all subjects.

### Carotid MR Imaging Protocol

Carotid MR imaging was performed on a whole-body 3.0T MR scanner (Achieva TX, Philips Healthcare, Best, The Netherlands) with the custom-designed 36-channel neurovascular coil. The carotid arteries were imaged using a multi-contrast vessel wall imaging protocol with the following sequences: (1) three-dimensional time-of-flight (3D TOF), (2) quadruple-inversion-recovery T1-weighted (T1W) imaging, (3) multislice double inversion-recovery T2-weighted (T2W) imaging, and (4) magnetization-prepared rapid acquisition gradient-echo (MP-RAGE). The TOF, T1W, T2W, and MP-RAGE images were acquired cross-sectionally centered at the bifurcation of index carotid arteries with a field of view 140 × 140 mm^2^. The slice thickness was 2 mm for T1W, T2W, and 1 mm for TOF and MR-RAGE imaging, respectively. The longitudinal coverage was 32 mm for T1W, T2W, and 48 mm for TOF and MP-RAGE, respectively. The imaging parameters are detailed in the Supplementary Material.

### MR Image Analysis

Carotid MR images were reviewed by two experienced radiologists (LD and ZX) who had >3 years of experience in vascular imaging with consensus blinded to clinical information using a custom-designed software of CASCADE (University of Washington, Seattle, Washington, United States). The image quality of each slice was rated using 4-point scales according to the clearness and signal-to-noise ratio of vessel wall boundaries and plaque components and the artifacts: 1, poor; 2, marginal; 3, good; and 4, excellent. MR images with image quality ≥2 were interpreted.

Carotid plaque was defined as eccentric wall thickening or having any plaque compositions such as calcification, LRNC, or IPH on MR imaging. The boundaries of carotid artery lumen and wall were traced manually and all carotid morphological characteristics (maximum wall thickness [Max WT], normalized wall index [NWI = wall area / total vessel area ×100%], luminal stenosis, etc.) that were representatives of plaque burden were measured. For patients with ischemic stroke, the carotid artery which is responsible for the symptoms was considered as the index side and included in the statistical analysis. For patients with TIA, the index artery was defined as lesions with high-risk plaque feature of large LRNC (occupied > 40% of wall area), IPH, and fibrous cap rupture (13) or larger Max WT bilaterally when there was no high-risk feature. The presence or absence of each plaque composition, such as calcification, LRNC, IPH, was determined by utilizing published criteria (13, 17). The luminal stenosis was measured using North American Symptomatic Carotid Endarterectomy Trial (18) criteria for extracranial carotid arteries.

### Follow-Up and Clinical Outcome

All patients were followed up for 1 year. The patients were systematically assessed and the outcomes were determined based on both clinical symptoms and imaging results. The clinical outcome was defined as any vascular event, such as ischemic stroke, TIA, or acute coronary syndrome. The cerebral infarct of anterior circulation was assessed on the brain using diffusion-weighted imaging or T2-Fluid-Attenuated Inversion Recovery images during follow-up. The acute coronary syndrome was determined by myocardial enzyme detection or coronary arteriography during follow-up. Two physicians and one trained nurse were blind to groups' information of patients and collected the clinical information by telephone or a face-to-face interview for routine medical outpatient clinic attendance and inquiring whether patients had experienced any vascular event in the past time. If patients did not respond to the follow-up, then we searched for their medical records and tried to contact their relatives for more information about the patient's condition. All outcome variables such as ischemic stroke, TIA, or acute coronary syndrome were evaluated with standard diagnostic guidelines by a physician or hospital medical records. All the information of follow-up patients can systematically be retrieved from national, regional, or hospital registers.

### Statistical Analysis

Count data were presented as frequencies or percentages. Numerical data in normal distribution were expressed as mean ± SD, while data with non-normal distributions were expressed as the median (interquartile range, IQR). The patients were divided into the following groups: co-existing hypertension and H-Hcy group; isolated hypertension group; isolated H-Hcy group; and control group (no hypertension and H-Hcy). Baseline clinical characteristics and carotid plaque features were compared between patient groups using independent *t*-test, Mann–Whitney *U*-test, or Chi-square (ANOVA) test. A *p* < 0.017 (0.05 divided by the times of comparisons) was considered statistically significant in multiple comparison analyses. For determining the predictors of the clinical outcome, univariate, and multivariate Cox proportional hazards regression functions were used to calculate the hazard ratio (HR) and the corresponding 95% confidence interval (CI) of possible determinants of vascular events, considering the time variable. The Kaplan–Meier product-limit method was used to estimate cumulative event-free rates in subgroups for graphical display depending on the presence of co-existing hypertension and H-Hcy or combination with carotid plaque features. Well-known risk factors and all clinical risk factors with *p* < 0.10 during univariate analysis were included in the multivariate models. Highly right-skewed variables were log-transformed or *Z-*score transformed before their inclusion in the regression models. The *p* < 0.05 was considered statistically significant except for the multiple comparison analysis. Statistical analysis was performed using SPSS v22.0 software (Statistical Package for the Social Sciences, International Business Machines, Inc., Armonk, New York, United States).

## Results

A total of 227 patients with recent cerebrovascular symptoms (<2 weeks) were screened from 323 community populations and enrolled for baseline MR scanning and clinical information acquisition, of which 10 patients were excluded due to poor image quality. Of the remaining 217 patients (mean age, 59.4 ± 11.9 years old; 154 males) with acceptable image quality, 82 (37.8%) were in co-existing hypertension and H-Hcy group, 69 (31.8%) were in the isolated hypertension group, 30 (13.8%) were in the isolated H-Hcy group, and 36 (16.6%) were in the control group, respectively. There were significant differences in gender, age, history of hyperlipidemia, HDL, Hcy, smoking, diabetes, and the treatment of anti-hypertension among four groups (all *p* < 0.05, Table 1).

**Table 1:** Clinical characteristics of the study population (*n* = 217).

		**Mean ±SD or n (%)**				***P*-value**
	**All patients** **(*n* = 217)**	**Co-existing hypertension and H-Hcy** **(*n* = 82)**	**Isolated hypertension** **(*n* = 69)**	**Isolated H-Hcy** **(*n* = 30)**	**Control group** **(*n* = 36)**	
Age, years	59.4 ± 11.9	61.2 ± 10.6	60.4 ± 10.4	57.8 ± 12.1	54.8 ± 15.6	0.039
Gender, male	154 (71.0)	65 (79.3)	39 (56.5)	25 (75.8)	25 (75.8)	0.007
BMI, kg/m^2^	25.3 ± 3.5	25.5 ± 3.5	26.0 ± 3.0	23.9 ± 3.7	24.8 ± 3.4	0.053
History of hyperlipidemia	100 (46.1)	44 (53.7)	28 (40.5)	16 (42.4)	12 (42.4)	0.042
HDL, mmol/l	1.1 ± 0.4	1.1 ± 0.4	1.2 ± 0.6	1.05 ± 0.2	1.02 ± 0.2	0.038
LDL, mmol/l	2.8 ± 1.2	2.9 ± 1.2	2.9 ± 1.4	2.9 ± 0.8	2.3 ± 0.9	0.055
TG, mmol/l	1.6 ± 0.9	1.7 ± 0.9	1.6 ± 0.8	1.5 ± 1.1	1.4 ± 0.8	0.480
TC, mmol/l	4.4 ± 1.2	4.5 ± 1.2	4.5 ± 1.2	4.5 ± 1.0	3.9 ± 1.0	0.058
Hcy, μmol/L	17.9 ± 11.0	23.2 ± 9.2	11.3 ± 2.1	26.4 ± 13.9	10.0 ± 3.3	<0.001
Stain use	104 (47.9)	45 (54.9)	28 (40.6)	16 (47.0)	15 (47.0)	0.096
Antihypertension treatment	114 (52.5)	57 (69.5)	50 (72.5)	0 (0)	0 (0)	0.001
History of smoking	128 (59.0)	58 (70.7)	32 (46.4)	22 (57.6)	16 (57.6)	0.002
History of diabetes	68 (31.3)	21 (25.6)	31 (44.9)	7 (24.2)	9 (24.2)	0.035
History of CHD	27 (12.4)	13 (15.9)	9 (13.4)	1 (7.6)	4 (7.6)	0.260

*BMI, body mass index; HDL, high-density lipoprotein; LDL, low-density lipoprotein; TG, Triglycerides; TC, Total cholesterol; Hcy, homocysteine; CHD, coronary heart disease*.

### Comparison of Carotid Plaque Characteristics Between Groups

The morphological and compositional features of carotid arteries are summarized in Table 2. Patients in co-existing hypertension and H-Hcy group had a significantly higher prevalence of carotid LRNC (73.2 vs. 50%, *p* = 0.015) than the control group. There were no significant differences in carotid plaque morphological and compositional characteristics between isolated hypertension group and control group, between isolated H-Hcy group and control group, and between co-existing hypertension and H-Hcy group and isolated hypertension group (all *p* > 0.017). Compared to patients in the isolated H-Hcy group, those in co-existing hypertension and H-Hcy group showed a significantly higher prevalence of carotid calcification (50.0 vs. 23.3%, *p* = 0.012) and LRNC (73.2 vs. 43.3%, *p* = 0.003). In addition, patients in the isolated hypertension group were found to have significantly greater carotid maximum wall thickness (3.4 ± 1.5 mm vs. 2.6 ± 1.1 mm, *p* = 0.005) compared to those in the isolated H-Hcy group.

**Table 2:** Comparison of carotid plaque features among different groups (*n* = 217).

	**Mean ±SD, median (IQR), or** ***n*** **(%)**				***P-*** **value**					
	**Co-existing hypertension and H-Hcy** **(*n* = 82)**	**Isolated hypertension** **(*n* = 69)**	**Isolated H-Hcy** **(*n* = 30)**	**Control group** **(*n* = 36)**	***P* ^a^**	***P* ^b^**	***P* ^c^**	***P* ^d^**	***P* ^e^**	***P* ^f^**
**Plaque morphology**										
Mean lumen area, mm^2^	44.2 ± 16.5	45.0 ± 14.9	45.3 ± 13.9	43.7 ± 14.0	0.982	0.809	0.714	0.689	0.483	0.781
Mean wall area, mm^2^	35.4 ± 12.4	33.6 ± 11.5	60.7 ± 7.5	33.0 ± 11.0	0.333	0.712	0.807	0.376	0.060	0.288
Mean total vessel area, mm^2^	79.6 ± 22.8	78.6 ± 20.0	76.0 ± 18.0	76.7 ± 17.0	0.632	0.619	0.961	0.944	0.617	0.812
Maximum wall thickness, mm	3.26 ± 1.6	3.4 ± 1.5	2.6 ± 1.1	3.3 ± 1.9	0.672	0.268	0.099	0.391	0.026^*^	0.005^*^
Mean NWI, %	45.9 ± 0.1	43.9 ± 0.1	42.4 ± 0.1	44.4 ± 0.1	0.289	0.898	0.678	0.115	0.074	0.620
Luminal stenosis, %	11.6 ± 23.8	18.5 ± 34.4	9.3 ± 25.8	20.5 ± 36.2	0.806	0.776	0.237	0.505	0.212	0.117
**Presence of plaque components**										
Calcification	41 (50)	27 (39.1)	7 (23.3)	12 (33.3)	0.095	0.561	0.375	0.183	0.012^*^	0.130
Lipid-rich necrotic core	60 (73.2)	44 (63.8)	13 (43.3)	18 (50.0)	0.015^*^	0.175	0.592	0.215	0.003^*^	0.060
Intraplaque hemorrhage	14 (17.1)	13 (18.8)	1 (3.3)	4 (11.1)	0.409	0.310	0.238	0.778	0.060	0.043^*^
**Volume of plaque components**										
Calcification	15.7 (4.8, 45.0)	16.5 (4.0, 43.3)	26.6 (4.5, 83.4)	11.6 (2.8, 41.6)	0.520	0.626	0.554	0.900	0.745	0.848
Lipid-rich necrotic core	62.0 (25.9, 211.8)	62.9 (33.6, 179.2)	33.5 (19.0, 72.8)	37.0 (20.3, 91.8)	0.134	0.612	0.936	0.906	0.158	0.098

a *Comparison between co-existing hypertension and H-Hcy group and control group;*

b *comparison between isolated hypertension group and control group;*

c *comparison between isolated H-Hcy group and control group;*

d *comparison between co-existing hypertension and H-Hcy group and isolated hypertension group;*

e *comparison between co-existing hypertension and H-Hcy group and isolated H-Hcy group;*

f *comparison between isolated hypertension group and isolated H-Hcy group.*

* *The volume comparison is applicable only for carotid arteries with the corresponding plaque component.*

### Association of Co-Existing Hypertension and H-Hcy With Subsequent Vascular Events

After baseline examination of 217 patients, 64 patients were excluded from the final association analysis due to loss of follow-up (*n* = 38) or underwent carotid endarterectomy and stenting (*n* = 26). Of 153 patients, the clinical information was collected from 31 patients and 122 patients by telephone and a face-to-face interview, respectively. Of 153 patients, 57 (37.3%), 51 (33.3%), and 17 (11.1%) had co-existing hypertension and H-Hcy, isolated hypertension, and isolated H-Hcy, respectively. During the median follow-up time of 12.2 ± 4.3 months, of 153 patients, 61 patients suffered from recurrent vascular events, including ischemic stroke (*n* = 19), TIA (*n* = 37), and acute coronary syndromes (*n* = 5). Clinical characteristics and carotid plaque features between patients with and without vascular events were compared and summarized in Table 3.

**Table 3:** Comparison of clinical characteristics and carotid plaque features in patients with and without subsequent vascular events (*n* = 153).

	**Mean ±SD or** ***n*** **(%)**		
	**Patients with vascular events** **(*n* = 61)**	**Patients without vascular events** **(*n* = 92)**	***P*-value**
**Clinical information**			
Age, years	60.2 ± 9.7	58.0 ± 13.3	0.341
Gender, male	45 (79.3)	63 (71.4)	0.483
BMI, kg/m^2^	25.9 ± 3.7	25.2 ± 3.1	0.147
Antihypertension treatment	39 (63.9)	40 (42.9)	0.008
History of hyperlipidemia	23 (37.7)	40 (42.9)	0.432
HDL, mg/dL	1.1 ± 0.6	1.1 ± 0.4	0.530
LDL, mg/dL	2.9 ± 1.6	2.7 ± 1.1	0.957
TG, mg/dL	1.5 ± 0.9	1.7 ± 1.0	0.336
TC, mg/dL	4.3 ± 1.3	4.3 ± 1.2	0.850
Hcy, μmol/L	18.1 ± 11.2	16.3 ± 10.5	0.119
Lipid lowering treatment	24 (39.3)	43 (46.4)	0.305
History of smoking	35 (57.4)	49 (56.0)	0.618
History of diabetes	14 (23.0)	28 (28.6)	0.292
History of CHD	7 (11.5)	10 (9.5)	0.907
H-Hcy hypertension	31 (50.8)	26 (28.3)	0.005
Isolated hypertension	18 (29.5)	33 (35.9)	0.415
Isolated H-Hcy	5 (8.2)	12 (13.0)	0.352
**Presence of plaque components**			
Calcification	29 (47.5)	28 (31.0)	0.052
Lipid-rich necrotic core	46 (75.4)	45 (47.6)	0.002
Intraplaque hemorrhage	9 (14.8)	12 (14.3)	0.915
High risk plaque	13 (21.3)	21 (28.6)	0.214

*BMI, body mass index; HDL, high-density lipoprotein; LDL, low-density lipoprotein; TG, triglycerides, TC, total cholesterol; Hcy, homocysteine; H-Hcy, hyper-homocysteine; CHD, coronary heart disease*.

Table 4 presented the results of Cox regression analysis. Univariate Cox regression analysis showed that co-existing hypertension and H-Hcy (HR, 1.97; 95% CI, 1.19–3.27; *p* = 0.009), presence of carotid LRNC (HR, 2.28; 95% CI, 1.27–4.09; *p* = 0.006), and combination of co-existing hypertension and H-Hcy and presence of carotid LRNC (HR, 2.44; 95% CI, 1.46–4.06; *p* = 0.001) were significantly associated with subsequent vascular events. After adjusting for baseline confounding factors including age, gender, basal metabolic index, antihypertension treatment, history of hyperlipidemia and diabetes, and presence of carotid calcification, the associations of co-existing hypertension and H-Hcy (HR, 1.82; 95% CI, 1.01–3.27; *p* = 0.44), presence of carotid LRNC (HR, 2.25; 95% CI, 1.09–4.65; *p* = 0.029) and combination of co-existing hypertension and H-Hcy and carotid LRNC (HR, 2.39; 95% CI, 1.26–4.43; *p* = 0.007) with subsequent vascular events remained statistically significant. Patients with a combination of existing hypertension and H-Hcy and carotid LRNC were more likely to develop subsequent vascular events compared to co-existing hypertension and H-Hcy and carotid LRNC alone.

**Table 4:** Cox regression hazard models of risk factors for vascular events.

	**Vascular events**					
	**Univariate regression**			Multivariate regression^*^		
	**HR**	**95% CI**	***P-*value**	**HR**	**95% CI**	***P-*value**
Hypertension	2.16	1.14–1.09	0.018^*^	1.00	0.38–2.64	0.966
H-Hcy	1.09	0.88–1.35	0.442	1.58	0.84–2.97	0.159
Presence of carotid LRNC	2.28	1.27–4.09	0.006^*^	2.25	1.09–4.65	0.029^*^
Co-existing hypertension and H-Hcy	1.97	1.19–3.27	0.009^*^	1.82	1.01–3.27	0.044^*^
Combination of co-existing hypertension and H-Hcy and carotid LRNC	2.44	1.46–4.06	0.001^*^	2.39	1.26–4.43	0.007^*^

* *Confounding factors: age, gender, BMI, antihypertension treatment, history of hyperlipidemia and diabetes, and carotid calcification. LRNC, lipid-rich necrotic core; HR, hazard ratio*.

**Figure 1:**
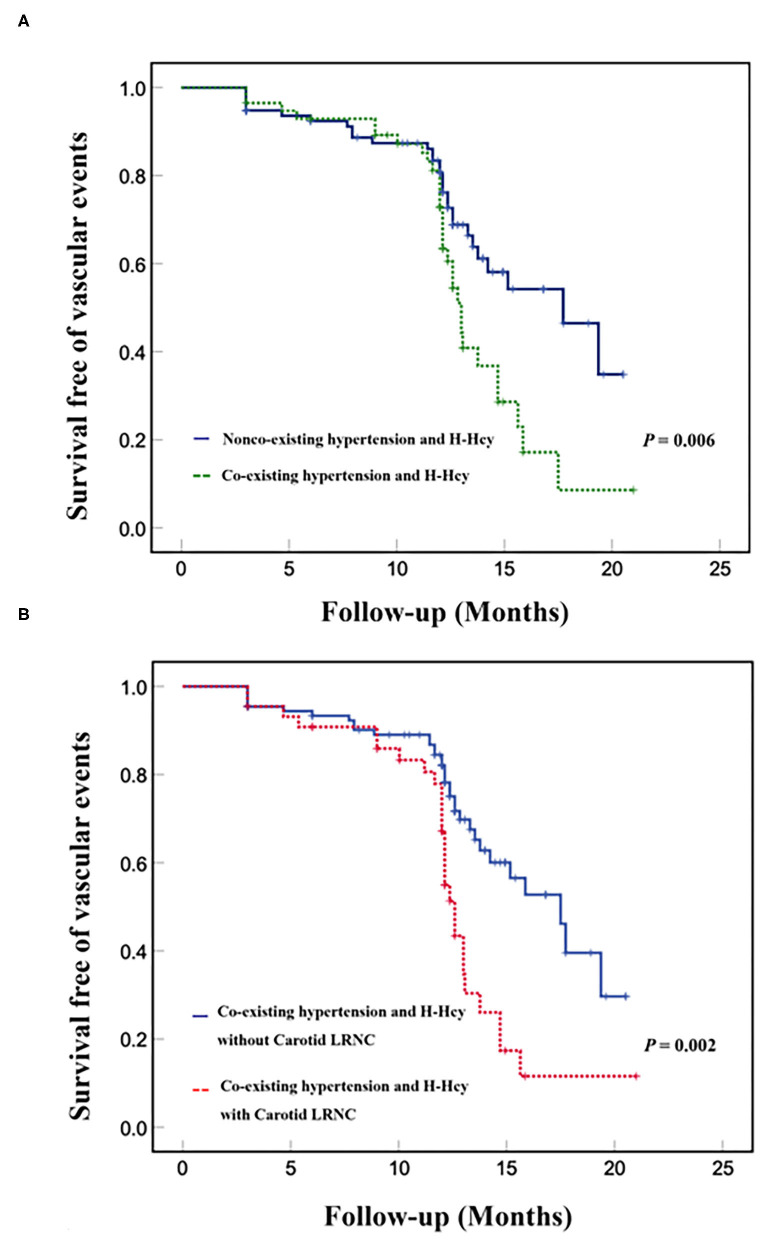
Kaplan-Meier analysis of survival free of vascular events in co-existing hypertension and H-Hcy. Kaplan-Meier curves of event-free survival of subsequent vascular events in patients with and without co-existing hypertension and H-Hcy **(A)**. Kaplan-Meier curves of event-free survival of subsequent vascular events in patients with co-existing hypertension and H-Hcy alone and patients with a combination of coexisting hypertension and H-Hcy with carotid LRNC **(B)**. The X-axis represents the time of follow-up in months. The Y axis represents the proportion of patients who were survival free of vascular events.

Kaplan–Meier curves for the incidence of subsequent vascular events showed that event-free survival was significantly higher for patients without co-existing hypertension and H-Hcy than those patients with co-existing hypertension and H-Hcy (*p* = 0.006, Figure 1A). Figure 1B showed that event-free survival was significantly higher for patients with co-existing hypertension and H-Hcy alone than those patients with a combination of co-existing hypertension and H-Hcy and carotid LRNC (*p* = 0.002).

**Figure 2:**
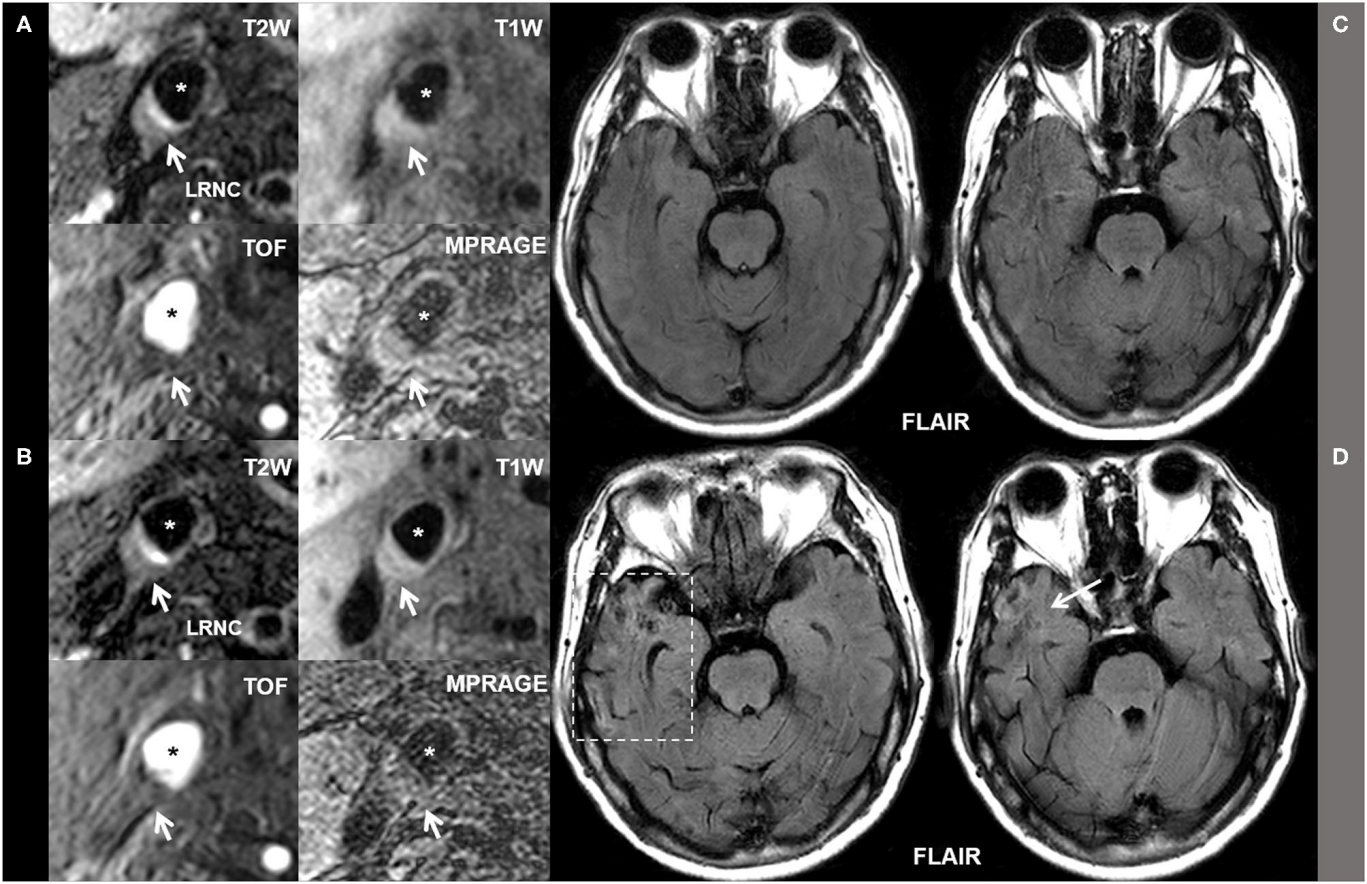
The baseline **(A,C)** and follow-up **(B,D)** MR images of a patient who had LRNC plaque in the right carotid with co-existing hypertension and H-Hcy (25 μmol/L). The LRNC (white arrows on T1W, T2W, TOF, and MP-RAGE images) in the right carotid artery and ipsilateral subsequent ischemic stroke (white arrows on Flair images) in the temporal lobe had progressed after 1-year follow-up. * Marks lumen of common carotid artery.

Figure 2 represents the baseline (Figures 2A,C) and follow-up (Figures 2B,D) MR images of a patient who had LRNC plaque in the right carotid with co-existing hypertension and H-Hcy (25 μmol/L). The LRNC (white arrows on T1W, T2W, TOF, and MP-RAGE images) in the right carotid artery and ipsilateral subsequent ischemic stroke (white arrows on Flair images) in the temporal lobe had progressed after 1-year follow-up.

## Discussion

To the best of our knowledge, this is the first prospective study to investigate the association of co-existing hypertension and H-Hcy with carotid vulnerable plaque characteristics, and the predictive value of the combination of co-existing hypertension and H-Hcy with carotid plaque features for subsequent vascular events using MR vessel wall imaging. We found that patients with co-existing hypertension and H-Hcy were more likely to have carotid LNRC compared to patients with isolated H-Hcy alone or those in the control group. In addition, we found that co-existing hypertension and H-Hcy, carotid LRNC, and a combination of co-existing hypertension and H-Hcy with carotid LRNC were independently predictive for subsequent vascular events. Higher HR value of a combination of co-existing hypertension and H-Hcy with carotid LRNC than each measurement alone indicates that a combination of traditional risk factors and carotid high-risk features could enhance the predictive value of each measurement alone for future events.

In the present study, patients with isolated hypertension were found to have a significantly greater carotid plaque burden of maximum wall thickness compared to those with isolated H-Hcy. We also found that patients with co-existing hypertension and H-Hcy showed marginally greater carotid maximum wall thickness than those with isolated H-Hcy. Since wall thickness measured by MR vessel wall imaging has been demonstrated to be associated with intima-media thickness measured by ultrasound in the common carotid artery (19), our results are consistent with previous ultrasound imaging studies. A retrospective study on Chinese patients with hypertension by Ye et al. (20) revealed that carotid intima-media thickness (CIMT) measured by performing ultrasound in patients with co-existing hypertension and H-Hcy was greater than that in patients with isolated H-Hcy (0.78 ± 0.26 mm vs. 0.68 ± 0.20 mm, *p* < 0.001). A Chinese community cohort study recruiting elderly subjects reported that the risk of increased CIMT in patients with hypertension and H-Hcy was significantly greater than other groups (OR, 1.67; 95% CI, 1.15–2.43) (21). Previous studies showed that patients with hypertension had increased CIMT due to changes of remodeling (22) and stiffness (23)of carotid artery compared to normotensive patients (0.487 ± 0.13 mm vs. 0.572 ± 0.141 mm, *p* < 0.001). Selhub et al. (24) demonstrated that high plasma homocysteine concentrations are associated with an increased risk of extracranial carotid-artery stenosis (OR, 2.0; 95% CI, 1.4–2.9) in the elderly through their role in homocysteine metabolism. Co-existing hypertension and H-Hcy will accumulate the risk of isolated hypertension and H-Hcy which may aggravate the progression of carotid plaque burden.

Our data showed that the prevalence of carotid LRNC is significantly higher in patients with co-existing hypertension and H-Hcy than that in patients with isolated hypertension group and control group. Most of the previous studies focused on the relationships between carotid morphology and H-Hcy and hypertension. Zhang et al. (25) reported that co-existing hypertension and H-Hcy was an independent risk factor for asymptomatic extracranial artery stenosis (relative risk: 3.16; 95% CI, 2.00–5.00). In addition, investigators found that patients with co-existing hypertension and H-Hcy were more likely to have occurrence (72.7 vs. 66 vs. 64.4%) and progression of carotid plaque (64.3 vs. 54.2 vs. 56.3%) compared to isolated hypertension and isolated H-Hcy patients (21, 26). It has been shown that presence of Hcy in atherosclerotic plaque can recruit macrophages, induce M0 macrophages to M1 (pro-inflammatory) polarization, increase inflammatory response, and induce macrophage mitochondrial damage and apoptosis, and finally lead to the formation of LRNC (27). Furthermore, hypertension can trigger or induce loss of vasomotor activity, perpetuate endothelial damage which might contribute to the inflammation and formation of foam cells in atherosclerotic plaque, thus indirectly enhance the opportunity of formation of LRNC (28–31). Hence, both high levels of Hcy in serums and hypertension can accelerate the progression of LRNC in carotid atherosclerotic plaque either by the indirect or direct pathway of pro-inflammatory. This can be also explained by the fact that the pro-inflammatory superimposing effect may be more pronounced than that of isolated H-Hcy and hypertension alone. Our study provides direct evidence for the important role of co-existing hypertension and H-Hcy in the occurrence and volume of LRNC which is a potential therapeutic target for carotid atherosclerosis. However, in the present study, no significant differences were found in the volume of LRNC and calcification among co-existing hypertension and H-Hcy, isolated hypertension, isolated H-Hcy, and control groups. Because the volume comparison was applicable only for carotid arteries with the corresponding plaque component, the valid sample of each group was limited which may attenuate the statistical power in multiple comparison analyses. Future studies with a larger sample size are suggested.

We found that both co-existing hypertension and H-Hcy and carotid LRNC were independently associated with subsequent vascular events. This finding is expected because the associations of carotid LRNC and co-existing hypertension and H-Hcy with subsequent cerebrovascular events have been well-evidenced in previous studies. A study by Kwee et al. (14) proved that the presence of carotid LRNC (HR, 3.20; 95% CI, 1.08–9.50; *p* = 0.036) was associated with the recurrence of cerebrovascular ischemic events in symptomatic patients with carotid atherosclerosis. Takaya et al. (32) reported that a larger maximum percentage of LRNC (HR for 10% increase, 1.6; *p* = 0.004) in patients with asymptomatic 50–79% carotid stenosis was an independent predictor for subsequent cerebrovascular events. Li et al. (15) have shown that the National Institute of Health stroke scale (NIHSS) score on admission in co-existing hypertension and H-Hcy group was significantly higher than that in the control group (6.32 ± 5.91 vs. 3.97 ± 3.59, *p* < 0.05) and patients in co-existing hypertension and H-Hcy group had the highest prevalence of subsequent stroke compared to other groups (22.83%) during the 1-year follow-up. A study by Zhong et al. (33) found that patients with co-existing hypertension and H-Hcy were at the highest risk of poor outcomes among all participants. Another study also revealed that co-existing hypertension and H-Hcy (OR, 2.988; 95% CI, 1.162–7.686) was an independent predictor for recurrence stroke (34). Co-existing hypertension and H-Hcy may promote vascular inflammation and oxidative stress, damage endothelial cells, inhibit endothelium-dependent relaxation and enhance thrombogenicity (35–38). Our findings also demonstrated that patients with both co-existing hypertension and H-Hcy and carotid LRNC had a higher risk of subsequent cerebrovascular events compared to those with each measurement alone, suggesting the combination of these two measurements could enhance the predictive value for future events.

This study has several limitations. First, this is a prospective study with a smaller sample size. Future studies with a larger sample size are suggested. Second, this study focused on the evaluation of the incremental value of atherosclerotic plaque features in extracranial carotid arteries for co-existing hypertension and H-Hcy in predicting subsequent vascular events. Recent studies have shown that co-existing intracranial and extracranial carotid artery atherosclerotic diseases are a stronger indicator for cerebrovascular events than atherosclerosis in each vascular bed alone. It is valuable to determine the predictive value of a combination of co-existing hypertension and H-Hcy and co-existing intracranial and extracranial carotid artery diseases in future studies. Third, the longitudinal coverage of the current carotid MR vessel wall imaging protocol is 32 mm centered to the carotid bifurcation. This coverage is limited for atherosclerotic lesions developed in more proximal or distal segments of carotid arteries. The 3D vessel wall imaging techniques (39) with larger longitudinal coverage is suggested to be utilized in future studies. Finally, the present study did not perform the carotid vessel wall imaging during follow-up. It will be valuable to investigate the relationship between the progression of carotid plaque and the vascular events in future studies.

## Conclusions

Co-existing hypertension and H-Hcy are associated with carotid vulnerable plaque features, particularly lipid-rich necrotic core. Combining co-existing hypertension and H-Hcy with carotid vulnerable plaque features has a stronger predictive value for subsequent vascular events than each measurement alone. Our findings suggest that it is vital to pay more attention to carotid plaque composition assessment in patients with co-existing hypertension and H-Hcy for stroke prevention.

## Data Availability Statement

The raw data supporting the conclusions of this article will be made available by the authors, without undue reservation.

## Ethics Statement

The studies involving human participants were reviewed and approved by Institutional Review Board of Tsinghua University. The patients/participants provided their written informed consent to participate in this study.

## Author Contributions

DL, HQ, and XZ contributed to the conception and design of the study. DL, HQ, JL, and XC collected the data. DL, HQ, XY, WD, and JS analyzed and interpreted the data. DL drafted the manuscript. HQ and XZ revised the manuscript. All authors contributed to the article and approved the submitted version.

## Funding

This study was supported by grants of the National Natural Science Foundation of China (Nos. 82171996, 81771825, and 81801694), the National Key R&D Program of China (2017YFC1307900 and 2017YFC1307904), the Key Areas Research and Development Program of Guangdong (2019B020235001), and the Guangdong Province Universities and Colleges Pearl River Scholar Funded Scheme (2017).

## Conflict of Interest

The authors declare that the research was conducted in the absence of any commercial or financial relationships that could be construed as a potential conflict of interest. The handling editor YZ declared a shared affiliation with several of the authors DL, XY, and JS at the time of review.

## Publisher's Note

All claims expressed in this article are solely those of the authors and do not necessarily represent those of their affiliated organizations, or those of the publisher, the editors and the reviewers. Any product that may be evaluated in this article, or claim that may be made by its manufacturer, is not guaranteed or endorsed by the publisher.
