# Supplementary material for: Co-existing Hypertension and Hyperhomocysteinemia Increases the Risk of Carotid Vulnerable Plaque and Subsequent Vascular Event: An MR Vessel Wall Imaging Study
Source: Front Cardiovasc Med. 2022 Mar 30;9:858066. doi: 10.3389/fcvm.2022.858066 (PMC9005821; doi:10.3389/fcvm.2022.858066)
Supplement: Supplementary file 1 [file Table_1.DOCX]

**SUPPLEMENTAL MATERIAL**

**Supplemental Tables**

Table 1. The imaging parameters for carotid artery MR vessel wall imaging.

|  | T1W | T2W | TOF | MP-RAGE |
| --- | --- | --- | --- | --- |
| Sequence | TSE | TSE | FFE | FFE |
| Black blood | QIR | MDIR | - | - |
| TR, ms | 800 | 4800 | 20 | 8.8 |
| TE, ms | 10 | 50 | 4.9 | 5.3 |
| Flip angle, deg | 90° | 90° | 20° | 15° |
| FOV, mm | 140×140 | 140×140 | 140×140 | 140×140 |
| In-plane resolution, mm^2^ | 0.5×0.5 | 0.5×0.5 | 0.5×0.5 | 0.5×0.5 |
| Slice thickness, mm | 2 | 2 | 1 | 1 |

TOF: time-of-flight, MP-RAGE: Magnetization Prepared Gradient Recalled Echo, CMRA: cardiovascular magnetic resonance angiography, TSE: turbo spin echo, FFE: fast field echo, QIR: quadruple inversion recovery, MDIR: multi-slice double inversion recovery. TR: repeat time, TE: echo time, FOV: field of view, T1W: T1 weighted, T2W: T2 weighted
